# The Associations between the Homeostatic and Circadian Sleep Processes and the Neurobehavioral Functioning (NBF) of Individuals with ADHD—A Systematic Review

**DOI:** 10.3390/brainsci13081134

**Published:** 2023-07-28

**Authors:** Reut Gruber, Gabrielle Gauthier-Gagné, Charlotte Little, Ziqi Fu

**Affiliations:** 1 Attention, Behaviour and Sleep Laboratory, Douglas Mental Health University Institute, Montréal, QC H4H 1R3, Canada; 2 Department of Psychiatry, McGill University, Montréal, QC H3A 0GA, Canada; 3 Integrated Program in Neuroscience, Faculty of Medicine, McGill University, Montréal, QC H3A 0G4, Canada

**Keywords:** attention-deficit/hyperactivity disorder (ADHD), sleep, homeostatic, circadian, neurobehavioral functioning, sustained attention, inhibitory control, working memory

## Abstract

The objective of the present review was to systematically examine associations between perturbations of the homeostatic or circadian sleep processes and the neurobehavioral functioning (NBF) of individuals with ADHD. Electronic databases were searched for articles published between December 2013 and March 2023. Studies were included if they used objective measures of NBF, used objective or subjective measures of sleep, and focused on individuals with ADHD. Ten studies met these inclusion criteria. Of these, eight studies found perturbations in the interplay between NBF and Process S or Process C, and three studies did not. The quality of the studies was degraded because they failed to address key factors that affect the sleep processes and by the presence of methodological weaknesses. Our review suggests that homeostatic and circadian sleep processes are associated with NBF in individuals with ADHD. However, to confirm the validity of this conclusion, future studies should examine or control for confounders and utilize experimental designs that allow causality to be inferred.

## 1. Introduction

Attention-deficit/hyperactivity disorder (ADHD) is one of the most commonly diagnosed disorders in childhood and adulthood. A diagnosis of ADHD is dependent on developmentally inappropriate symptoms of inattention, hyperactivity, and/or impulsivity that persist for at least 6 months in two or more settings and are present before the age of 12 years [1,2,3]. Many individuals with a history of childhood ADHD continue to be impaired by the disorder in adulthood, although they often show reduced hyperactivity and impulsivity while retaining symptoms of inattention [4]. ADHD is found in 5.9% of youth [5] and 4.4% of adults aged 18 to 44 years. The persistence of ADHD leads to a wide range of emotional, educational, and social adjustment outcomes [6,7]. If untreated, individuals with ADHD struggle with impairments across many crucial domains of functioning, including academic, occupational, and social realms [2]. Brain differences in frontal–subcortical neural circuitry underlying motor control, executive functions, inhibition of behavior, and the modulation of reward pathways have been documented in individuals with ADHD compared with individuals without ADHD [8]. These differences are related to neurobehavioral challenges, including weaknesses in working memory, response inhibition, and planning/organization [9].

Inadequate sleep has been reported in up to 70% of children with ADHD [10,11,12,13,14,15,16,17], compared with 20% to 30% of children in the general population [18]. The sleep problems experienced by children with ADHD include unhealthy lifestyle choices, such as excessive evening screen use or insufficient sleep [19,20,21], behavioral problems (e.g., difficulties initiating and/or maintaining sleep) [22,23], and primary sleep disorders, such as sleep-disordered breathing (SDB), obstructive sleep apnea (OSA), restless leg syndrome (RLS), delayed sleep phase syndrome (DSPS), insomnia, and narcolepsy [24,25,26,27,28,29,30].

A two-process model is postulated to regulate sleep: Process S, a homeostatic, history-dependent process that regulates sleep duration and depth by creating sleep pressure as the wake time lengthens; and Process C, a circadian history-independent process that is driven internally by physiological cycles that rise and fall across the 24 h day. Process C determines sleep–wake timing and modulates the homeostatic drive for sleep and waking alertness [31] by generating a sleep–wake propensity rhythm. As the homeostatic sleep pressure intensifies, Process C intensifies wake-promoting signals that peak just before the habitual sleep episode [32]. Fluctuations in daytime sleepiness reflect circadian variations [33] in alertness [34].

A diagnosis of ADHD has been linked to insufficient and/or poorer-quality sleep [35,36], which negatively affects homeostatic sleep regulation (Process S). In addition, compared with typically functioning individuals, those with a diagnosis of ADHD are more likely to show a delay in the phase of sleep–wake timing, stronger evening preference [37], and significant variations in the daily rhythms of behavioral, cognitive, endocrine, physiological, and molecular processes [38]. These changes all reflect perturbation of the circadian sleep process (Process C).

Process S and Process C interact to affect cognitive performance [32], and homeostatic and circadian disturbances impede neurobehavioral functioning. Attention, working memory, and executive functions [39], which are the key domains exhibiting weaknesses among individuals with ADHD, are directly and strongly affected by both homeostatic pressure and circadian rhythmicity. An increase in the homeostatic sleep drive degrades performance on tasks measuring attention, cognitive speed, and memory; and causes wake-state instability. Endogenous circadian rhythmicity that persists even under conditions of chronic sleep deprivation [33] affects attention, working memory, and executive functions.

Despite the high prevalence of disturbed homeostatic and circadian sleep processes among individuals with ADHD and the critical roles of these processes in neurobehavioral functioning, no review has been published using the two-process model of sleep [31] to examine the neurobehavioral deficits of individuals with ADHD. This is a problem because without such knowledge clinicians who assess, diagnose, and treat individuals with ADHD will not be able to address sleep processes that may contribute to the very symptoms they are attempting to diagnose and treat.

The objective of the present review was to systematically examine the associations between perturbations of the homeostatic or circadian sleep processes and the neurobehavioral functioning (NBF) of individuals with ADHD. It was hypothesized that: (1) perturbation of slow-wave activity (SWA), non-REM sleep (NREM), theta activity during wakefulness, sleep duration, and wake after sleep onset (WASO) are associated with poorer NBF; (2) a higher level of daytime sleepiness or a performance period that misaligns with the circadian preference is associated with poorer NBF.

## 2. Materials and Methods

Literature search. A comprehensive review of the PubMed, Ovid MEDLINE, Ovid EMBASE, and SCOPUS databases was conducted based on Preferred Reporting Items for Systematic Reviews and Meta-Analyses (PRISMA) guidelines [40]. We used the terms ADHD, Attention Deficit*, COGNI, Executive Function*, Attention, Sleep, Circadian, DLMO, and Melatonin* as keywords or applied MeSH terms in combination with Boolean operators to maximize search sensitivity. Initial screening and study selection were performed using Rayyan, which is a web and mobile application for systematic reviews (for full search information, see Supplementary Materials).

The utilized inclusion criteria were the following: (1) peer-reviewed, (2) published between December 2013 and March 2023, (3) English language, (4) reported associations between sleep processes and objectively measured NBF, and (5) focused on individuals with ADHD. Studies that used subjective NBF measures, qualitative studies, case studies, conference abstracts, unpublished dissertations, and reviews were excluded.

Initial study selection was performed by three of the authors and discrepancies were resolved by open discussion among all authors. Screening involved review of titles and abstracts, followed by full texts. Forwards/backwards citation analysis of full texts was used to identify studies that had not been captured in the search. At least two reviewers (GGG, CL, and/or ZF) completed screening. An additional reviewer (RG) adjudicated disagreements to achieve consensus.

Data extraction and outcomes. Data were extracted from each article using a standard electronic form developed for this review. The form extracted the following: author and year; participant characteristics, including age in years (mean/median, standard deviation, range), sex (male/female, percentage), diagnoses, and medication use; sample size (total N); study design (qualitative/quantitative/both, cross-sectional); methodology (sleep, circadian, cognitive measures); and findings discussed in the results section relating to the associations between sleep/circadian processes and objectively measured cognitive outcomes. Detailed descriptions of the study characteristics and results are provided in Table 1.

Risk of bias determination. Three reviewers (GGG, CL, and ZF) assessed the methodological quality of the studies with the Mixed Methods Appraisal Tool (MMAT) (see Table 1). This quality appraisal tool can be applied to multiple designs (i.e., randomized, non-randomized, descriptive, and mixed methods). For each study design, five items can be appraised as yes, no, or unclear. Three review authors (GGG, ZF, CL) independently evaluated each included study, and the inter-rater agreement was 89%. Discrepancies between the two evaluators were resolved by consensus or, where agreement could not be reached, in consultation with another author (RG).

## 3. Results

**Figure 1:**
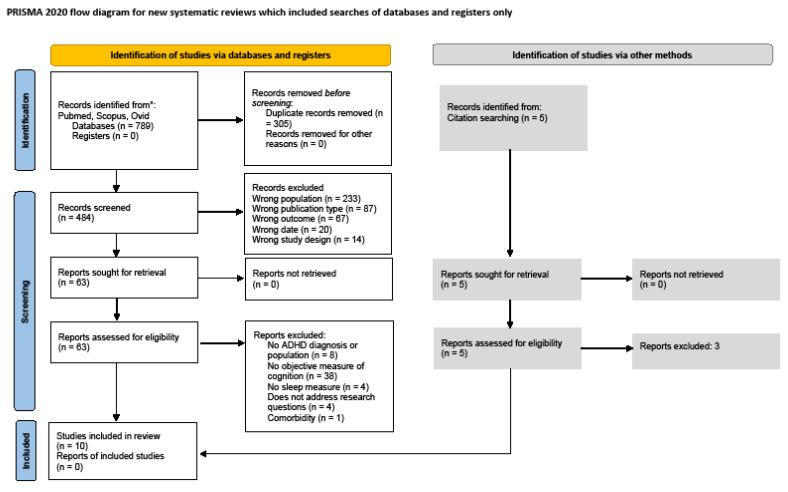
Legend: PRISMA: Preferred Reporting Items for Systematic Reviews and Meta-Analyses. Database search yielded 789 abstracts. Removal of duplicates resulted in 484 abstracts. Manual searches identified 5 additional studies, of which 3 were excluded. Blind double screening of the 484 abstracts resulted in 63 studies reviewed. An amount of 55 studies were excluded based on full-text review. In total, 10 studies were included.

Study selection. Figure 1 shows results derived from the Preferred Reporting Items for Systematic Reviews and Meta-Analyses (PRISMA) flow diagram. After the removal of duplicate records, the search identified 484 unique records for title and abstract screening. On these records, the evaluators had a 98% agreement rate on whether to retain records for full-text screening, with a disagreement on 11 records. After further discussion, it was decided that none of the disagreed-upon records would advance to full-text screening. In total, 63 records met the initial criteria and advanced to full-text screening. Of the 63 records, 10 met the full criteria for data extraction and were retained for the systematic review [64,65,66,67,68,69,70,71,72,73]. As seen in Figure 1, the most common reason for study exclusion was a lack of objective cognitive measures. Details of the 10 included studies are provided in Table 1.

Study characteristics. Characteristics of each of the 10 included studies are listed in Table 1.

Age. The included studies were conducted on children (*n* = 6), adolescents (*n* = 1), and adults (*n* = 3) diagnosed with ADHD. Seven studies included a control group (*n* = 534) and three included only participants with ADHD (*n* = 114).

Sample size. Of the 10 included studies, sample sizes ranged from 14 to 363 participants. In total, 320 participants with ADHD and 534 healthy controls (389 females, 453 males) were included. Four studies included only male participants, and the other studies included 57% females (*n* = 389) to 43% males (*n* = 289).

Indicators of the two sleep processes. Sleep Process S: The examined indicators of Process S included slow-wave activity (S) WA/theta) [67,68,70,72], sigma power [65], and sleep time spent awake during the night [19,66,69,71,73]. Process C: The examined indicators were time of day [65,67,69], chronotype [69], and daytime sleepiness [64,70]. The evidence showing that fluctuations in daytime sleepiness, alertness, and related impairments correspond to Process C is based on multiple lines of evidence including (1) studies that kept subjects continuously awake in a 72 h temporal isolation experiment with constant conditions, uniform food intake, and no knowledge of clock time. These studies revealed that the endogenous circadian oscillator responsible for body temperature rhythm is also a determinant of subjective alertness and that it changed in a way that was consistent with the documented circadian variation in human performance efficiency (e.g., [74]); (2) studies that have demonstrated significant associations between neurobehavioral performance deficit patterns following sleep restriction and core body temperature rhythm phase (e.g., [75]) [76,77,78]; (3) studies demonstrating that the degree of daytime sleepiness and impairments in performance due to sleep restriction were driven by phase angle difference between DLMO and sleep (i.e., sleeping and waking at a different phase of the circadian cycle); (4) studies that have shown that individual differences in sleepiness were driven by circadian phase even when assessed following acute sleep deprivation (e.g., [79]); and (5) studies that have shown significant associations between higher levels of sleepiness and later timing of melatonin onset [80].

Study designs. Nine of the studies used cross-sectional designs, allowing them to test associations between sleep parameters and NBF among individuals with ADHD. In addition, four experimental studies were conducted to examine the role of sleep parameters in NBF: two of the studies manipulated sleep duration by extending [64] or restricting [64,66] participants’ sleep. One study increased slow-wave oscillation by using transcranial direct-current stimulation and compared performance between a pre-manipulation performance session in the evening and a session in the morning following sham or stimulation conditions [72]. One study examined learning-induced increases in SWA by comparing SWA before and after daytime learning [68]. Finally, four studies examined circadian parameters by comparing participants’ performance at different times of day [67,69] or by examining associations between NBF and daytime sleepiness [64,70].

Domains of neurobehavioral functioning (NBF). The included studies measured sustained attention, inhibitory control, and working memory.

Sustained attention. Sustained attention was defined as the ability to sustain attention on a task for a period of time [81,82].

Inhibition control. Inhibition control was defined as a cognitive process that permits an individual to inhibit their impulses or dominant behavioral responses to stimuli in order to select more appropriate behaviors that are consistent with the individual’s goals.

Working memory. Working memory was defined as the processes used for temporarily storing and manipulating information in the face of ongoing processing and distraction [83].

### 3.1. Tasks Used to Operationalize NBF

Sustained attention. Sustained attention was operationalized using the following methods: (1) different versions of the continuous performance task (CPT; Ref. [65], including alertness, Ref. [69]; Conners’ continuous performance task (CPT, [47,48,84]) Ref. [70]; Sustained Attention to Response Task [50] Refs. [64,70]; the cued continuous performance task with flankers [47,48]; and the oddball task [66,70]). CPT tasks involve the sequential presentation of stimuli, letters, numbers, or visual targets over an extended period of time. The participant is asked to respond to particular target stimuli while ignoring other stimuli serving as nontarget distractors. The measured variables include reaction time (RT, associated with the subject’s ability to perform fast), reaction time variability (RTV, the subject’s stability in performance), inattention (omission errors, defined as the number or percentage of targets not responded to), and disinhibition commission errors, defined as the number or percentage of responses to stimuli other than the target. (2) a single reaction task [72], for which the average reaction time and the variability of the reaction time were calculated.

Inhibition control. Inhibition control was operationalized using the following methods: (1) the Matching Familiar Figures Test (MFFT [60,61], as used in [71,73], respectively), in which the participant is presented with a series of visual designs and a number of to-be-matched designs and is asked to correctly match the target with its identical mate within the group of designs. Failure to slow down combined with increased errors is taken as a failure of inhibitory control; (2) the go/no go task ([41,85]), as used in [67,72]), in which the participant is presented with randomly alternating stimuli and instructed to respond when they see the A stimulus but not the B stimulus. Here, A is presented more often to create a response set or prepotency toward responding, and errors in responding to B are taken as an index of failed inhibitory control.

Working memory. Working memory was operationalized using the N-back task [86,87] as used in ([69]), which asks participants to remember stimulus sequences, such as letters or pictures, during an ongoing secondary task, and judge whether each item matches the one presented N items ago. The processing load is manipulated by changing the value of N ([86,87]).

Quality Assessment. Limitations in the studies included a small sample size (100%); cross-sectional designs (9, 82%); and lack of control of the following confounding factors: daytime sleepiness (6, 54%%), time of day (9, 82%), circadian preferences (10, 91%), phase angle during performance (0, 100%), and sleep timing (10, 91%).

### 3.2. The Associations between Process S and Neurobehavioral Functioning (NBF)

EEG indices of Process S and NBF. A total of five studies examined EEG indices of Process S and NBF: one in preschool children [67], three in school-age children/adolescents [65,72,73], and one in adults [70]. The findings from these studies are summarized below per each age group:

Preschool Children. SWA and theta activity were significantly elevated in children with ADHD during REM sleep but not during NREM sleep, compared to controls [67]. Among typically developing preschool children, REM theta activity at frontal electrode sites was associated with better performance on a morning inhibitory control task. In preschool children with ADHD [67], there was no significant association between NBF and any sleep parameter.

School-Age Children. One experimental study [72] used transcranial oscillating direct current stimulation (toDCS) at 0.75 Hz to increase slow oscillations during deep sleep bilaterally over the pre-frontal cortex (PFC) in school-age/early adolescent children with ADHD. This led to an improvement in inhibitory control and did not change performance on sustained attention measured by CPT.

School-age Children/Adolescents. One study conducted with school-age children [65] found a positive association between performance on a sustained attention task and sigma power over the right frontal (F4) region in healthy children, whereas no significant correlation between sigma power and sustained attention was found in the ADHD group.

Another study conducted with school-age/adolescent participants with ADHD [68] found that although the performance of participants with ADHD and normally developing peers did not differ during a pre-sleep learning session of visuomotor information, participants with ADHD had no corresponding experience-dependent increase in SWA in the night following the learning session.

Adults. A study comparing EEG activity during a resting-state eyes-open paradigm in adults with ADHD and neurotypical adults [70] found that EEG slowing (calculated as the ratio of slow-frequency bands (delta + theta) to fast-frequency bands (alpha + beta)) was higher in the ADHD group versus the neurotypical group in all regions and was highest in the frontal region of the ADHD group. EEG slowing was a significant predictor of ADHD diagnostic status and symptom severity.

Associations between Process C indices—sleep duration or time spent awake during the night and NBF. Four studies examined the associations between NBF and sleep duration or time spent awake during the night. Two cross-sectional studies were conducted with school-age children [71,73], while two studies employed experimental changes of sleep duration in adolescents [64] or adults [66]. The findings from these studies are summarized below per each age group:

School-Age Children. Longer wake after sleep onset (WASO) and a higher actigraphy fragmentation index were associated with poorer inhibitory control [71]. A longer sleep period was associated with lower inhibitory control, whereas a longer stage 2 (N2 sleep) period and longer REM sleep latency were associated with better inhibitory control [73]. Another study conducted with school-age children found marginal but non-significant associations between performance on a CPT task and CSHQ measures of sleep duration and parasomnia and actigraphy-based measures of time in bed and sleep latency.

Adolescents. Sustained attention during a sleep-extension week was compared to that during a sleep-restriction week, with a 1.6 h difference in sleep duration between the weeks. Under the sleep-extension condition, WASO and sleep onset latency were longer and sleep efficiency was lower compared to the sleep-restriction week. No significant effect was found when CPT performance indices were compared between the sleep-restriction and sleep-extension week [64].

Adults. Acute sleep deprivation (continuous wakefulness for 25 h) increased commission and omission errors and reaction time variability in individuals with ADHD but not in the control group [66].

### 3.3. Process C and NBF

Process C-time of day and NBF. Three studies, one each in preschool children [67], school-age children [65], and adults [69], examined the impact of time of day on NBF in individuals with ADHD. The findings from these studies are summarized below per each age group:

Preschool children. Morning performance on sustained attention and inhibitory control was significantly better relative to baseline (evening) in typically developing children but not in children with ADHD, who showed no difference between the morning and baseline results [67].

School-age children. A sustained attention (alertness) task was completed four times: In a PSG condition, it was assessed in the evening before sleep and in the morning after sleep. In a second condition, it was assessed first in the morning and then in the evening of the same day. The conditions were assessed at least 2 weeks apart. No difference in performance was found between the different times of day [65].

Adults. Sustained attention, working memory, and daytime sleepiness in the morning and evening were examined among evening-chronotype young adults with and without ADHD [69]. There was a group-by-time interaction on one measure of sustained attention: the difference in reaction time for sustained attention between a morning and evening session was significantly greater in participants with an evening chronotype and ADHD compared to those with an evening chronotype and no ADHD. Participants in both groups performed better in the evening on measures of sustained attention and working memory [69].

### 3.4. Process C, Daytime Sleepiness and NBF

Children. No available studies.

Adolescents. No available studies.

Adults. One study examined associations between NBF and daytime sleepiness in adults [70]. Participants with ADHD were rated as sleepier than neurotypical participants based on observer-rated sleepiness during performance on the CPT. A higher level of observer-rated sleepiness was associated with a higher level of omission errors on the CPT used in the study (SART).

## 4. Discussion

The reviewed evidence suggests that perturbation of sleep processes contributes to NBF deficits in individuals with ADHD. Perturbations were identified among indicators of the homeostatic process, including observation of higher SWA and theta activity during REM sleep [65] and EEG slowing in the frontal region among adults with ADHD compared to healthy controls [70]. The associations between nighttime sleep and daytime performance were found to differ in children with ADHD versus controls. Sigma power was associated with performance on a measure of sustained attention in typically developing children but not in children with ADHD [65]. In addition, in contrast to healthy participants, those with ADHD showed no experience-dependent increase in SWA after the night following a learning experience undertaken the preceding night [68].

Some discrepancies can be seen in studies assessing whether sleep duration is associated or marginally associated with NBF. Some reports showed that sleep deprivation impaired NBF and a longer habitual sleep duration was associated with better NBF [66,71], whereas others failed to detect these associations [64,73]. This discrepancy could reflect that although participants in the latter set of studies had a longer sleep duration, they exhibited low sleep efficiency, longer duration of WASO, longer sleep latency [64], or a higher level of limb movement with arousal [73], which might have degraded the overall benefit of extended sleep on NBF. In addition, the findings were consistent with our hypothesis that a higher sleepiness level and misalignment of performance time with circadian preference were associated with poorer NBF. Enhancement of slow oscillations during deep sleep over the PFC improved the NBF of children with ADHD [72]. Together, the included studies suggest that the relationships between Processes S and C and NBF may differ in individuals with ADHD versus neurotypical individuals, and that amelioration of these processes has the potential to be used as a means to improve NBF in this population. Significant weaknesses were noticed across all of the included studies.

A number of parameters known to affect the sleep processes and NBF were not measured or controlled for in the included studies (see Table 1 for details on each included study), including circadian preference and performance time of day, phase angle, daytime sleepiness developmental changes, and physiological indices of the circadian system.

Circadian preference interacts with performance time, such that individuals perform better when the performance time is aligned with their circadian preference. For example, a person with a morning chronotype has a cognitive ability that is better in the morning hours and vice versa. In addition, performance suffers when the time of day is misaligned with the circadian preference. Only one of the included studies addressed chronotype and time of day in the context of group-level differences in NBF.

The phase angle is the relationship between the sleep episode and the circadian phase. Homeostatic sleep markers differ when measured in different circadian phases, but none of the included studies examined or controlled for the phase angle. Thus, the group-level differences identified between individuals with ADHD and controls on indicators of homeostatic sleep processes could reflect differences of the phase angle during the measurement rather than the diagnostic status per se.

Daytime sleepiness is known to affect NBF [75], but only one study integrated daytime sleepiness into its hypothesis testing.

Developmental changes: although age-related changes in Processes S and C are well documented [88], no study examined developmental differences in the interactions of Process S and Process C with NBF.

Physiological indices of the circadian system: none of the included studies examined the associations between NBF and physiological indices of the circadian system, such as dim-light melatonin onset (DLMO) or core body temperature.

Future studies should examine the potential impacts of performance time relative to circadian preference, phase angle, and daytime sleepiness on NBF in individuals with ADHD. In addition, future studies should use a consistent phase angle when assessing sleep and NBF, as this would enable researchers to differentiate between the contributions of homeostatic and circadian parameters to the NBF of individuals with ADHD [89]. They should also include a wider range of age groups and utilize longitudinal designs to obtain a comprehensive and precise characterization of the interplay among Process S and NBF. Furthermore, future studies seeking to reveal associations between circadian rhythmicity and the NBF of individuals with ADHD should use physiological markers of the endogenous circadian day and night in conjunction with objective measures of NBF.

### 4.1. Methodological Weaknesses

The majority of the studies included small samples that might have contributed to type II errors [90]. Future studies should seek to recruit larger populations of well-characterized participants. In addition, the use of cross-sectional designs in a number of studies [64,65,66,67,70,72] means that it is not possible to make causal inferences or further interpret the results regarding the impact of processes S and C on NBF. Future studies should employ experimental or longitudinal designs that allow causality to be determined when examining the impact of the two sleep processes on NBF among individuals with ADHD.

### 4.2. Clinical Implications

Findings from the included studies show that perturbation of homeostatic and circadian sleep processes could contribute to, exacerbate, or underlie NBF deficits. Hence, increasing clinicians’ knowledge of the ways in which sleep may affect cognitive functioning in individuals with ADHD could have far-reaching benefits. It is important that clinicians will be aware that the time of day of testing and the patient’s circadian preference, bedtime, wake-up time, sleep schedule, and sleep duration prior to testing may all affect test performance and potentially limit or even invalidate the interpretation of tests results if not considered. Ideally, these parameters should be standardized, monitored, and considered when interpreting results and making clinical recommendations based on the results. In addition, clinicians should routinely assess, monitor, and manage the sleep problems of patients with ADHD. In cases where circadian or homeostatic factors contribute to poor performance, such factors should be addressed or treated prior to starting the use of medications and in parallel with initiating educational interventions, with the goal of optimizing the collective impacts of these interventions.

### 4.3. Limitations and Future Directions

The designs and outcomes of the studies included in this review were too diverse to yield a meaningful summary estimate of effect. It is expected that the research gaps and limitations identified in this review will facilitate the design of future studies that yield findings that are suitable for integration in a meta-analysis. Such an analysis can be used to elucidate the contributions of Process S and Process C to the neurobehavioral functioning of individuals with ADHD.

## 5. Conclusions

The study found evidence for associations between perturbation of the homeostatic and circadian sleep processes and NBF in individuals with ADHD. The presence of multiple confounders, small sample sizes, and the use of cross-sectional designs lowered the quality of the evidence generated by the included studies. Future studies examining the interplay among Process S, Process C, and NBF should seek to ameliorate these limitations. Clinically, the findings highlight the importance of assessing sleep and circadian parameters when diagnosing and treating the NBF of individuals with ADHD.

## Figures and Tables

**Table 1:** Associations between perturbations of homeostatic or circadian sleep processes and NBF of individuals with ADHD of different age groups.

		Participant Characteristics			Design	Assessment of Sleep Problems or Disorders	Process S	Process C	Cognition	Results
		Inclusion Criteria	Age M yrs	Medications			Dimension(s); Measures		Dimension (s); Measures	
Associations between perturbations of homeostatic or circadian sleep processes and Sustained Attention and Inhibition in individuals with ADHD of different age groups										
Children										
44	ADHD DISC-IV [2]	ADHD: 6.70 TD: 6.73	ADHD grp: 1 Tenex, 1 Adderall	Cross-sectional	Phone interview (NR)	Sleep duration, sleep EEG; PSG	NA	Inhibitory control, sustained attention; go/no go task [41]	ADHD > TD: frontal theta and SWA during REM TD but not ADHD Positive associations between inhibitory control and REM frontal theta activity Improved performance following sleep	
42	DSM-5 [2] ADHD (K-SADS-PL [42])	ADHD: 10.7 TD children: 10.9 TD Adults: 24.95	ADHD grp: 5 MPH	Cross-sectional	Child-reported sleep questionnaire (NR), adult PSQI [43]	Sleep duration, timing, sleep EEG; PSG	Time of day	Sustained attention; computer-based vigilance test (NR)	TD vs. ADHD Significantly different correlation between parietal absolute sigma power and IQ Positive correlation between frontal absolute sigma power and alertness.	
Adolescents										
41	ADHD DSM-5 [2] (K-SADS PL [42]), IQ > 70 (KBIT-2 [44]),	15.10	ADHD grp: stimulant: 73.6%, non-stimulant: 6.9%, melatonin: 1.4%, summer washout	Within-subject crossover	SDB, RLS (PSQ [45])	Sleep duration, latency, efficiency, continuity; actigraphy, PDSS [46], daily logs	Daytime sleepiness	Sustained attention, inhibition control; CPT [47,48]	Sleep extension vs. restriction: (only ADHD group is included) No change in sustained attention, inhibition control	
Adults										
47	ADHD DSM-5 [2]; ADHD Severity (CAARS [49])	ADHD: 33.5 TD: 29.5		Cross-sectional	NA	Sleepiness; observer-rated sleepiness, EEG slowing during resting-state eyes-open	NA	Sustained attention; SART [50] CPT-OX [47,48]	ADHD > TD EEG slowing Observed sleepiness Sleepy ADHD > non-sleepy ADHD, TD Sustained attention, omission errors, reaction time	
43	ADHD DSM-IV (DISC-IV [51])	ADHD: 24.7 Control: 26.	ADHD grp: 24 h washout	Between groups repeated measures	PLM, OSA, RLS (MSQ [52], clinical interview, PSQI [51])	Sleep duration; actigraphy, sleep diary	NA	Sustained attention, inhibition control; oddball task		
Associations between perturbations of homeostatic or circadian sleep processes and Sustained Attention/Working Memory in individuals with ADHD of different age groups										
Children										
NA										
Adolescents										
NA										
Adults										
46	ADHD with ASRS [53] > 4. TD with ASRS < 4. Evening chronotype (rMEQ [54] < 12).	Phase 1. 19.5 Phase 2. 20.4	ADHD grp: stimulant medication washout night prior to/day of testing.	Cross-sectional	PSQI [43]	Sleep quality, duration; PSQI [43], ESS [55], SSS [56], sleep diary	Chronotype, time of day, daytime sleepiness	Working memory, executive functions, sustained attention; VSWM task [48], Stroop Task [57,58], CPT-II [47,48]	Time of day main effect Sustained attention and working memory performance better in the evening vs. morning (main effect beyond group) ADHD vs. TD ADHD longer reaction time in the morning vs. TD	
Associations between perturbations of homeostatic or circadian sleep processes and Inhibition/Impulsivity in individuals with ADHD of different age groups										
Children										
49	DSM-IV-R [2] ADHD (K-SADS-PL [42])	ADHD 12.3	ADHD grp: MPH: 10, 48 h washout	Randomized double-blind cross-over	Self-report	Sleep duration, quality, efficiency, sleep EEG; PSG	NA	Inhibition control, sustained attention; go/no go task (KiTAP, [59])	so-tDCS vs. sham (only ADHD group is included) Slow oscillation power increased Shorter RT and smaller RTSD on go/no-go task	
48	ADHD DSM-IV-TR [2] (clinical interview)	ADHD: 8.7 TD: 9.3	Medication naïve	Cross-sectional	Clinical interview (NR)	Sleep duration, continuity; Actigraphy	NA	Impulsivity; MFFT-K [60,61]	ADHD Response error rate was associated with actigraphy WASO and sleep fragmentation index Response latency rate was associated with sleep fragmentation index.	
50	ADHD DSM-IV [2] (clinical interview)	6–12 M = NR	Medication naïve	Cross-sectional	Clinical interview, PSG	Sleep duration, continuity, sleep EEG; PSG	NA	Impulsivity; MFFT-K [60,61]	ADHD Verbal IQ positively associated with SWS, limb movement index, and negatively with S2. Response error positively associated with sleep period time, limb movement index. Response latency time negatively correlated with S2, REM sleep latency.	
Adolescents										
NA										
Adults										
NA										
Associations between perturbations of homeostatic or circadian sleep processes and Learning in individuals with ADHD of different age groups										
Children										
45	ADHD DSM-IV [2] (clinical interview)	ADHD: 12.4 TD: 12.3	ADHD grp: MPH: 7, 1 did not take it the day of the sleep assessment.	Cross-sectional	NA	Deep sleep, SWA; PSG	NA	Left hemisphere (baseline) vs. local SWA increase in right hemisphere	ADHD vs. TD Local change in SWA is smaller in ADHD compared to controls	

*Note.* Process S: Homeostatic sleep process. Process C: Circadian sleep process. ADHD: Attention-deficit/hyperactivity disorder. TD: Typically developing. >: greater than. <: less than. NR: Not reported. TIB: Time in bed. TST: Total sleep time. S1: Stage 1 sleep. S4: Stage 4 sleep. PLM: Periodic limb movement. RLS: Restless leg syndrome. OSA: Obstructive sleep apnea. RT: Reaction time. RTSD: Reaction time variability. SWA: Slow-wave activity. REM: Rapid eye movement. NREM: Non-rapid eye movement. MPH: Methylphenidate. WASO: Wake after sleep onset. So-tDCS: Transcranial direct current stimulation. PSG: Polysomnography. EEG: Electroencephalogram. K-SADS-PL [42]: Schedule for Affective Disorders and Schizophrenia for School-Age Children-Present and Lifetime Version. KBIT-2 [44]: Kaufman Brief Intelligence Scale, Second Edition. PSQ [45]: Pediatric sleep questionnaire. PDSS [46]: Pediatric Daytime Sleepiness Scale. CPT: Continuous performance test. IQ: Intelligence quotient. PSQI [43]: Pittsburgh Sleep Quality Index. DISC-IV [51]: Diagnostic Interview Schedule for Children. MSQ [52]: Mini sleep questionnaire. ASRS [53]: ADHD-Adult Self-Report Scale. rMEQ [54]: Morningness–eveningness questionnaire-reduced. ESS [55]: Epworth Sleepiness Scale. SSS [56]: Stanford Sleepiness Scale. SART: Sustained Attention to Response Task. CAARS [49]: Conners’ Adult ADHD Rating Scales. CPT-OX [47,48]: Cued continuous performance task with flankers. DSM-IV [2]: Diagnostic and Statistical Manual—5th Edition. DSM-IV-TR [62]: Diagnostic and Statistical Manual of Mental Disorders, Fourth Edition, Text Revision. MFFT-K [60,61]: Matching Familiar Figures Test for Korean Children. K-WISC-III [63]: Korea Wechsler Intelligence Scale for Children. KiTAP [59]: Kinder-Testbatterie fur Aufmerksamkeitsprufung. ADHD grp: ADHD group.

## Data Availability

No new data were created.

## Supplementary Materials

The following supporting information can be downloaded at: https://www.mdpi.com/article/10.3390/brainsci13081134/s1.
