# Supplementary material for: The Associations between the Homeostatic and Circadian Sleep Processes and the Neurobehavioral Functioning (NBF) of Individuals with ADHD—A Systematic Review
Source: Brain Sci. 2023 Jul 28;13(8):1134. doi: 10.3390/brainsci13081134 (PMC10452539; doi:10.3390/brainsci13081134)
Supplement: Supplementary file 1 [file brainsci-13-01134-s001.zip › brainsci-2412967-supplementary.pdf]

Supplementary Materials

Literature Search

This Supplementary Materials outlines the literature search. The three databases accessed were Pubmed from the United States National Library of Medicine at the National Institutes of Health, Scopus from Elsevier, and Ovid MEDLINE from the National Library of Medicine. The abstracts of the initial hits were downloaded from each database (see tables 1, 2 and 3 below for search details) in .ris format to the digital reference manager EndNote. Pubmed (43 hits), Scopus (405 hits), and Ovid (341 hits). Total: 789 hits. The hits were deduplicated using the Find Duplicates function on EndNote for a total of 549 hits. The resulting hits were uploaded to Rayyan, a web-tool for systematic review, and 65 additional duplicates were removed using Rayyan's Detect Duplicates Tool for a total of 484 hits. Two blinded reviewers screened each hit for inclusion using the title and abstract for guidance. Discrepancies in inclusion decisions were discussed to determine a common decision.

Table S1. Search syntax and results from PubMed database.

#1 – Sleep & Circadian, accessed on 27 February 2023
((ADHD[Title/Abstract]) OR (Attention Deficit*[Title/Abstract])) AND (Cogni*[Title/Abstract]) AND ((Executive Function*[Title/Abstract]) OR (Attention[Title/Abstract])) AND ((Sleep[Title/Abstract]) OR (Circadian[Title/Abstract])) Filters: Books and Documents, Classical Article, Clinical Study, Clinical Trial, Clinical Trial, Phase I, Clinical Trial, Phase II, Clinical Trial, Phase III, Clinical Trial, Phase IV, Controlled Clinical Trial, Evaluation Study, Government Publication, Introductory Journal Article, Meta-Analysis, Multicenter Study, Observational Study, Pragmatic Clinical Trial, Preprint, Randomized Controlled Trial, Twin Study, Validation Study, in the last 10 years, Humans, English.
Results: 31
#2 – DLMO & ADHD, accessed on 12 Mar 2023
(("DLMO"[All Fields] OR ("melatonin"[MeSH Terms] OR "melatonin"[All Fields] OR "melatonin s"[All Fields] OR "melatonine"[All Fields] OR "melatonins"[All Fields])) AND ("attention deficit disorder with hyperactivity"[MeSH Terms] OR ("attention"[All Fields] AND "deficit"[All Fields] AND "disorder"[All Fields] AND "hyperactivity"[All Fields]) OR "attention deficit disorder with hyperactivity"[All Fields] OR "adhd"[All Fields])) AND ((y_10[Filter]) AND (clinicalstudy[Filter] OR clinicaltrial[Filter] OR controlledclinicaltrial[Filter] OR introductoryjournalarticle[Filter] OR observationalstudy[Filter] OR preprint[Filter] OR randomizedcontrolledtrial[Filter]) AND (fft[Filter]) AND (humans[Filter]) AND (english[Filter]))
Results: 12
#3 – DLMO & ADHD & Cognition, accessed on 15 Mar 2023
(("DLMO"[All Fields] OR ("melatonin"[MeSH Terms] OR "melatonin"[All Fields] OR "melatonin s"[All Fields] OR "melatonine"[All Fields] OR "melatonins"[All Fields]))
AND ("attention deficit disorder with hyperactivity"[MeSH Terms] OR ("attention"[All Fields] AND "deficit"[All Fields] AND "disorder"[All Fields] AND "hyperactivity"[All Fields]) OR "attention deficit disorder with hyperactivity"[All Fields] OR "adhd"[All Fields]))
AND ("cognition"[All Fields] OR ("cognition"[MeSH Terms])) AND ((y_10[Filter]) AND (clinicalstudy[Filter] OR clinicaltrial[Filter] OR controlledclinicaltrial[Filter] OR introductoryjournalarticle[Filter] OR observationalstudy[Filter] OR preprint[Filter] OR randomizedcontrolledtrial[Filter]) AND (fft[Filter]) AND (humans[Filter]) AND (english[Filter]))
Results: 0
Total Results: 43

Table S2: Search syntax and results from Scopus database

#1 – Sleep & Circadian, accessed on 27 February 2023
TITLE-ABS (((adhd) OR (attention AND deficit*)) AND (cogni*) AND ((executive AND function*) OR (attention)) AND ((sleep) OR (circadian))) AND PUBYEAR > 2012 AND PUBYEAR < 2024 AND (LIMIT-TO (DOCTYPE , "ar")) AND (LIMIT-TO (LANGUAGE , "English")) AND (LIMIT-TO (EXACTKEYWORD , "Human"))
Results: 283
#2 – DLMO & ADHD, accessed on 12 Mar 2023
TITLE-ABS ((dlmo OR melatonin) AND (adhd)) AND (LIMIT-TO (DOCTYPE , "ar")) AND (LIMIT-TO (LANGUAGE , "English"))
Results: 71
#3 – DLMO & ADHD & Cognition, accessed on 15 Mar 2023
TITLE-ABS ((dlmo OR melatonin) AND (adhd) AND (cogni*)) AND (LIMIT-TO (DOCTYPE , "ar")) AND (LIMIT-TO (LANGUAGE , "English"))
Results: 51
Total Results: 405

Table S3: Search syntax and results from Ovid Database

#1 – Sleep & Circadian, accessed on 27 February 2023
(TI=(((ADHD) OR (Attention Deficit*)) AND (Cogni*) AND ((Executive Function*) OR (Attention)) AND ((Sleep) OR (Circadian)))) OR (AB=((((ADHD) OR (Attention Deficit*)) AND (Cogni*) AND ((Executive Function*) OR (Attention)) AND ((Sleep) OR (Circadian)))))
Filter: Language: English, Publication Type: Article or Early access
Results: 341
Total Results: 341
